# Development and validation of a predictive model for American Society of Anesthesiologists Physical Status

**DOI:** 10.1186/s12913-019-4640-x

**Published:** 2019-11-21

**Authors:** Seshadri C. Mudumbai, Suzann Pershing, Thomas Bowe, Robin N. Kamal, Erika D. Sears, Andrea K. Finlay, Dan Eisenberg, Mary T. Hawn, Yingjie Weng, Amber W. Trickey, Edward R. Mariano, Alex H. S. Harris

**Affiliations:** 1 Anesthesiology and Perioperative Care Service, Veterans Affairs Palo Alto Health Care System, 3801 Miranda Avenue, Palo Alto, CA 94402 USA; 2 Department of Anesthesiology, Perioperative and Pain Medicine, Stanford University School of Medicine, 291 Campus Drive, Stanford, CA 94305 USA; 3 Center for Innovation to Implementation, Veterans Affairs Palo Alto Health Care System, 795 Willow Road (152-MPD), Menlo Park, California 94025 USA; 4 Department of Ophthalmology, Stanford University School of Medicine, 291 Campus Drive, Stanford, CA 94305 USA; 5 Department of Orthopaedic Surgery, Stanford University School of Medicine, 291 Campus Drive, Stanford, CA 94305 USA; 6 Department of Surgery, Section of Plastic Surgery at the University of Michigan, 2101 Taubman Center 1500 E. Medical Center Drive, Ann Arbor, MI 48109 USA; 7 Center for Clinical Management Research, VA Ann Arbor Healthcare System, 2215 Fuller Rd, Ann Arbor, MI 48105 USA; 8 Department of Surgery Surgery Policy Improvement Research and Education (S-SPIRE) Center, Stanford University School of Medicine, 291 Campus Drive, Stanford, CA 94305 USA

**Keywords:** Anesthesiology/methods, Health status, Clinical prediction rule, Outcome assessment (health care), Reliability and validity

## Abstract

**Background:**

The American Society of Anesthesiologists Physical Status (ASA-PS) classification system was developed to categorize the fitness of patients before surgery. Increasingly, the ASA-PS has been applied to other uses including justification of inpatient admission. Our objectives were to develop and cross-validate a statistical model for predicting ASA-PS; and 2) assess the concurrent and predictive validity of the model by assessing associations between model-derived ASA-PS, observed ASA-PS, and a diverse set of 30-day outcomes.

**Methods:**

Using the 2014 American College of Surgeons National Surgical Quality Improvement Program (ACS NSQIP) Participant Use Data File, we developed and internally cross-validated multinomial regression models to predict ASA-PS using preoperative NSQIP data. Accuracy was assessed with C-Statistics and calibration plots. We assessed both concurrent and predictive validity of model-derived ASA-PS relative to observed ASA-PS and 30-day outcomes. To aid further research and use of the ASA-PS model, we implemented it into an online calculator.

**Results:**

Of the 566,797 elective procedures in the final analytic dataset, 8.9% were ASA-PS 1, 48.9% were ASA-PS 2, 39.1% were ASA-PS 3, and 3.2% were ASA-PS 4. The accuracy of the 21-variable model to predict ASA-PS was C = 0.77 +/− 0.0025. The model-derived ASA-PS had stronger association with key indicators of preoperative status including comorbidities and higher BMI (concurrent validity) compared to observed ASA-PS, but less strong associations with postoperative complications (predictive validity). The online ASA-PS calculator may be accessed at https://s-spire-clintools.shinyapps.io/ASA_PS_Estimator/

**Conclusions:**

Model-derived ASA-PS better tracked key indicators of preoperative status compared to observed ASA-PS. The ability to have an electronically derived measure of ASA-PS can potentially be useful in research, quality measurement, and clinical applications.

## Background

The American Society of Anesthesiologists Physical Status Classification system (ASA-PS) is a commonly used, subjective method to categorize patients’ fitness for surgery [1, 2]. Originally developed by Saklad et al., the six-point classification system ranges from healthy patients with no comorbidities (ASA-PS 1) to brain-dead patients whose organs are being removed for donor purposes (ASA-PS 6) [3]. Though the system was initiated more than 5 decades ago, the scoring system continues to perform fairly well in assessing patients for both inpatient and outpatient surgery [4].

While the original intent of the ASA-PS was to stratify severity of illness prior to surgery, more recently the ASA-PS has been used as a simple means to predict outcomes [5–7]. While other and potentially better surgical outcome prediction methods are available, most have been developed for specific surgical conditions rather than for ‘surgery’ in general [8]. The ASA-PS has face validity as an assessment of functional capacity, which is increasingly thought to be a significant predictor of patient outcome [9]. ASA-PS is now included within risk-adjustment algorithms comparing hospital performance in surgical care i.e., the American College of Surgeons National Surgical Quality Improvement Program (ACS NSQIP) [10]. Models drawn entirely from preoperative NSQIP data may also be particularly helpful in risk stratification during the preoperative evaluation process [7, 11, 12].

Though the ASA-PS has validity as a marker of patients’ preoperative health status, multiple studies nevertheless have found that inter-rater reliability is moderate, meaning different anesthesiologists often give the same patient different classification levels [2, 13, 14]. Studies also indicate that ASA-PS may be missing or misclassified in data registries, which can lead to miscalculations of outcomes benchmarking for facilities [15, 16]. Most concerning are ASA-PS scores that are far from their expected value given observed patient characteristics – for example a patient with a ASA-PS IV but no recorded comorbidities. While ASA-PS as recorded in clinical databases may have measurement errors, an automated, risk model-derived calculation of ASA-PS that takes into account multiple aspects of the patients condition can serve as an initial proxy for improving and evaluating quality of care. The automated, risk model-derived ASA-PS may suggest more accurate initial values or corrections to these errors. Accordingly, the objectives of this study were to 1) develop and internally cross-validate a predictive model for ASA-PS using a wide range of preoperative predictors; and 2) assess the concurrent and predictive validity of the model by assessing associations between predicted ASA-PS, observed ASA-PS, and a diverse set of 30-day outcomes.

## Methods

### Data sources

We used de-identified registry data; therefore, the study was exempt from IRB review. Our data source was the 2014 Participant Use File (PUF) of the American College of Surgeons National Surgical Quality Improvement Program (ACS-NSQIP), which is available to member institutions [17]. The ACS-NSQIP collects data on over 150 variables, including preoperative risk factors, ASA-PS, intraoperative variables, and 30-day postoperative mortality and morbidity outcomes for patients undergoing major surgical procedures in both the inpatient and outpatient surgical setting. Definitions for the variables are found within the PUF file.

### Development of analytic sample

**Fig. 1:**
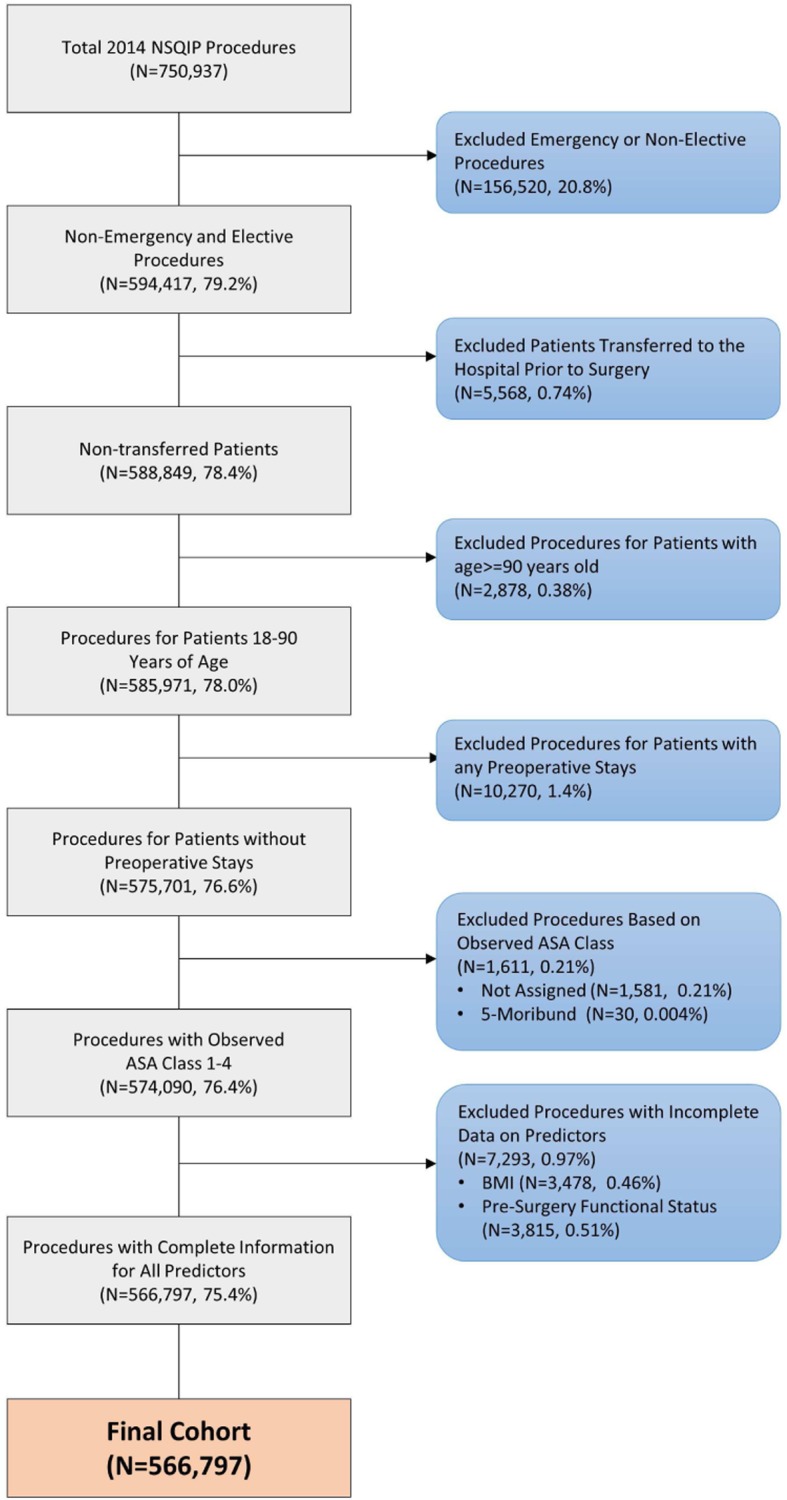
Flowchart of Cohort Inclusion and Exclusion Criteria

From the 750,937 surgeries represented in the 2014 PUF(Fig. 1), we excluded procedures that met any of the following criteria: emergent or non-elective surgeries; surgeries for patients transferred to the hospital (not admitted from home); patients < 18 years old or ≥ 90 years old at the time of surgery; procedures for patients who were inpatients immediately prior to their index surgery; surgeries for patients who had missing ASA assignment or had a ASA-PS class of ‘5-Moribund’; and procedures of patients who had incomplete data for any of the predictors identified for model development (see below). We selected these criteria given evidence of lower inter-rater agreement for ASA-PS for emergent cases or at extremes of age [14]. In addition, in trauma surgery, interrater reliability for assigning ASA-PS is fair at best and complete information on preoperative variables may be missing [18].

### Development and internal validation of a model to predict ASA-PS

#### Outcome

Clinical assignment of ASA-PS status as represented in the 2014 NSQIP PUF on a scale from 1 to 4 (Table 1).

**Table 1:** Current Definitions and Examples of American Society of Anesthesiologists Physical Status (ASA-PS) Class

ASA Physical Status Classification	Definition	Examples, including, but not limited to:
ASA-PS I	A normal healthy patient	Healthy, non-smoking, no or minimal alcohol use
ASA-PS II	A patient with mild systemic disease Mild diseases only without substantive functional limitations.	Mild diseases only without substantive functional limitations. Examples include (but not limited to): current smoker, social alcohol drinker, pregnancy, obesity (30 < BMI < 40), well-controlled DM/HTN, mild lung disease
ASA-PS III	A patient with severe systemic disease	Substantive functional limitations; One or more moderate to severe diseases. Examples include (but not limited to): poorly controlled DM or HTN, COPD, morbid obesity (BMI ≥40), active hepatitis, Alcohol dependence or abuse, implanted pacemaker, moderate reduction in ejection fraction, ESRD undergoing regularly scheduled dialysis, premature infant PCA < 60 weeks, history (> 3 months) of MI, CVA, TIA, or CAD/stents.
ASA-PS IV	A patient with severe systemic disease that is a constant threat to life	Recent (< 3 months) history of MI, CVA, TIA, or CAD/stents. Ongoing cardiac ischemia or severe valve dysfunction, severe reduction of ejection fraction, sepsis, DIC, ARD or ESRD not undergoing regularly scheduled dialysis
ASA-PS V	A moribund patient who is not expected to survive without the operation	Ruptured abdominal/thoracic aneurysm, massive trauma, intracranial bleed with mass effect, ischemic bowel in the face of significant cardiac pathology or multiple organ/system dysfunction
ASA-PS VI	A declared brain-dead patient whose organs are being removed for donor purposes	

Available at www.asahq.org/resources/clinical-information/asa-physical-status-classification-system; The addition of “E” denotes emergency surgery: (An emergency is defined as existing when delay in treatment of the patient would lead to a significant increase in the threat to life or body part)

#### Predictors

We identified predictors that would be commonly available during preoperative evaluation such as demographics, codes indicative of common diagnoses and treatments, and functional status before surgery. We also evaluated preoperative laboratory data (i.e., pre-operative serum sodium) as potential predictors and noted that they were missing for a large proportion of cases, most likely not at random; and so did not include them in our final model.

- Sex: Defined as either male or female
- Race/Ethnicity: Defined with 6 categories: non-Hispanic White; non-Hispanic Black; Hispanic; non-Hispanic Asian; non-Hispanic Other; Unknown
- Age: Age of patient (18–89 yrs)
- Body mass index (BMI): Defined by height and weight by the following formula; (703 x wt (lbs) / (ht (in)) ^2^
- Diagnoses and Treatments:
  - ◦ diabetes (None, insulin dependent; non-insulin dependent);
  - ◦ hypertension required medication (yes, no; The patient must have been receiving or required long-term treatment of their chronic hypertension for > 2 weeks.);
  - ◦ chronic obstructive pulmonary disease (yes, no);
  - ◦ congestive heart failure (yes, no);
  - ◦ renal failure (yes, no);
  - ◦ disseminated cancer (yes, no);
  - ◦ smoker (yes, no);
  - ◦ sepsis (none vs. any sepsis; septic shock; or systemic inflammatory response syndrome (SIRS);
  - ◦ ascites (yes, no);
  - ◦ preoperative wound infection (yes, no);
  - ◦ weight loss (yes, no);
  - ◦ bleeding disorders (yes, no);
  - ◦ dyspnea (none; at rest; or with moderate exertion);
  - ◦ the presence of mechanical ventilation greater than 48 h prior to surgery (yes, no);
  - ◦ smoking status prior to surgery (yes, no);
  - ◦ bleeding prior to surgery (yes, no),
  - ◦ dialysis (yes, no);
  - ◦ steroid use (yes, no);
  - ◦ transfusion (yes, no);
  - ◦ functional status before surgery (independent; partially dependent; totally dependent to perform activities of daily living [ADLs] in the 30 days prior to surgery).

#### Statistical analysis

Multinomial logistic regression was fitted on our final sample using ASA-PS as the outcome and variables listed above as predictors. The model was fitted using ‘nnet’ package built in R 3.3.0 [19]. We internally validated the model performance using 10-fold cross-validation. The C-statistic, an index of model accuracy, was calculated as the mean across the 10 repetitions. The C-Statistic can be defined as the probability that a person who has been clinically assigned to a specific ASA classification has a higher predicted probability of being in that class than someone with a different classification [20, 21]. The C-statistic and its corresponding 95% CI was calculated using the ‘pROC’ package in R. We compared the concurrent validity of predicted vs. observed ASA-PS (i.e., patients with predicted ASA-PS > observed ASA-PS vs. those with observed ASA-PS > predicted ASA-PS) by examining group associations with BMI, comorbidities, and functional status using two-sample Wilcoxon rank-sum (Mann-Whitney) tests.

### Assessing the predictive validity of the ASA-PS model

#### Outcomes

As defined in the ACS-NSQIP PUF, 30-day postoperative complications included the following binary (yes/no) outcome variables: mortality; and morbidity: venous thromboembolism; deep incisional surgical site infection; cardiac complications [myocardial infarction], cerebrovascular accident; respiratory complications [respiratory failure]; wound infections; sepsis; returned to the OR; renal insufficiency; and any complication.

#### Predictors

Coefficients from the multinomial logistical regression were used to predict the probabilities of each ASA-PS class (1-no disturbance, 2-mild, 3-severe, 4-life threat) for each person in the sample. We then assigned an ASA-PS class to each person based on their highest predicted probability (the class most likely) outputted from the model. In addition to the predicted ASA score, the following potential intraoperative confounders were included: Current Procedural Terminology (CPT) body system group (primary procedure codes were classified by major organ system type i.e., gastrointestinal, musculoskeletal etc., using Clinical Classification Software [CCS] systems); wound classification (clean/contaminated; contaminated; dirty/infected); anesthesia type (general anesthesia; spinal; epidural; monitored anesthesia care (MAC); or unknown); hospital length of stay in days; and operative time (total operative duration in hours) [22].

#### Statistical analysis

To compare the predictive power of observed vs. predicted ASA class, we first conducted separate logistic regressions using either as the independent variable and each 30-day postoperative complication as the outcome. C-statistics and the corresponding bootstrapped 95% CI in each model were calculated and compared by the two ASA class types in parallel. In addition, we compared the C-statistics for each outcome adjusting for important intraoperative variables such as CPT body system group, wound classification, anesthesia type, hospital length of stay and operation type. We then further evaluated the predictive validity of our model by conducting a patient-level pairwise analysis i.e., a contingency table of predicted ASA-PS vs. observed ASA-PS for each outcome (presence vs. absence).

We used SAS software, version 9.24 (SAS Institute Inc., Cary, NC, USA) and R software, version 3.0.2 (https://www.r-project.org/) for the statistical analyses, online tool development and graphics. To aid further research and use of the ASA-PS model, we implemented an online calculator using Shiny [23].

## Results

### Sample characteristics

Our final analytical dataset included a total of 566,797 elective procedures (Fig. 1 and Table 2). Overall, most of the patients (88%) were ASA-PS 2 (48.9%) or 3 (39.1%), followed by ASA 1 (8.9%) and ASA 4 (3.2%). On average, the patients were in their mid-50s. The majority were female, obese (class I), predominantly white, mostly non-diabetic, hypertension requiring medications, with about one-fifth being smokers. Most of the patients did not have a diagnosis of COPD, CHF, renal failure, disseminated cancer, ascites, wound infections, weight loss, require transfusions, or having been on a ventilator.

Table 3 provides the most frequent procedures by CPT-body system classification and Top 20 level-one CCS (mapped by CPT) codes. The majority of patients had a digestive or musculoskeletal system procedure, though there were representative procedures across all other organ systems. The most frequent specific surgeries included those classified as other, hernia repairs, hysterectomy, and colorectal resections.

**Table 2:** Characteristics of Study Sample

	All		ASA-PS ^a^1		ASA-PS 2		ASA-PS 3		ASA-PS 4	
	*N* = 566,797		50,285	8.9%	277,156	48.9%	221,453	39.1%	17,903	3.2%
	Avg	sd	Avg	sd	Avg	sd	Avg	sd	Avg	sd
Age	55.9	15.6	40.5	14.1	53.5	14.8	61.7	13.8	66.5	12.6
BMI	30.5	7.7	26.5	4.8	29.5	6.4	32.7	8.8	31.2	9.5
	no.	%	no.	%	no.	%	no.	%	no.	%
*n=*	566,797		50,285	8.9%	277,156	48.9%	221,453	39.1%	17,903	3.2%
Sex										
Female	329,669	58.2%	28,288	56.3%	171,512	61.9%	122,624	55.4%	7245	40.5%
Male	237,128	41.8%	21,997	43.7%	105,644	38.1%	98,829	44.6%	10,658	59.5%
Race/Ethnicity										
White	376,372	66.4%	26,273	52.2%	181,892	65.6%	155,687	70.3%	12,520	69.9%
Black	51,757	9.1%	3502	7.0%	23,248	8.4%	22,967	10.4%	2040	11.4%
Hispanic	37,982	6.7%	4499	8.9%	19,779	7.1%	12,749	5.8%	955	5.3%
Asian	14,675	2.6%	2127	4.2%	8103	2.9%	4228	1.9%	217	1.2%
Other	5156	0.9%	420	0.8%	2487	0.9%	2135	1.0%	114	0.6%
Unknown	80,855	14.3%	13,464	26.8%	41,647	15.0%	23,687	10.7%	2057	11.5%
Dyspnea										
No	535,798	94.5%	50,049	99.5%	270,904	97.7%	200,913	90.7%	13,932	77.8%
At Rest	1517	0.3%	12	0.0%	226	0.1%	954	0.4%	325	1.8%
Moderate Exertion	29,482	5.2%	224	0.4%	6026	2.2%	19,586	8.8%	3646	20.4%
Diabetes										
No	484,620	85.5%	50,085	99.6%	257,655	93.0%	165,458	74.7%	11,422	63.8%
Non-Insulin	54,427	9.6%	159	0.3%	15,501	5.6%	35,767	16.2%	3000	16.8%
Insulin	27,750	4.9%	41	0.1%	4000	1.4%	20,228	9.1%	3481	19.4%
Sepsis										
None	564,736	99.6%	50,176	99.8%	276,369	99.7%	220,454	99.5%	17,737	99.1%
Any Sepsis	338	0.1%	14	0.0%	126	0.0%	167	0.1%	31	0.2%
Septic Shock	10	0.0%	1	0.0%	0	0.0%	6	0.0%	3	0.0%
SIRS^b^	1713	0.3%	94	0.2%	661	0.2%	826	0.4%	132	0.7%
Functional Status										
Independent	561,050	99.0%	50,238	99.9%	276,133	99.6%	217,680	98.3%	16,999	95.0%
Partially Dependent	5321	0.9%	47	0.1%	976	0.4%	3484	1.6%	814	4.5%
Totally Dependent	426	0.1%	0	0.0%	47	0.0%	289	0.1%	90	0.5%
Steroid Use										
No	547,932	96.7%	50,114	99.7%	271,015	97.8%	210,236	94.9%	16,567	92.5%
Yes	18,865	3.3%	171	0.3%	6141	2.2%	11,217	5.1%	1336	7.5%
Hypertension with Medications										
No	312,618	55.2%	49,344	98.1%	183,331	66.1%	76,345	34.5%	3598	20.1%
Yes	254,179	44.8%	941	1.9%	93,825	33.9%	145,108	65.5%	14,305	79.9%
Dialysis										
No	562,089	99.2%	50,284	100.0%	277,011	99.9%	218,794	98.8%	16,000	89.4%
Yes	4708	0.8%	1	0.0%	145	0.1%	2659	1.2%	1903	10.6%
COPD^c^										
No	544,439	96.1%	50,252	99.9%	274,216	98.9%	205,483	92.8%	14,488	80.9%
Yes	22,358	3.9%	33	0.1%	2940	1.1%	15,970	7.2%	3415	19.1%
Ascites										
No	565,885	99.8%	50,278	100.0%	276,930	99.9%	220,878	99.7%	17,799	99.4%
Yes	912	0.2%	7	0.0%	226	0.1%	575	0.3%	104	0.6%
Congestive Heart Failure (CHF)										
No	564,691	99.6%	50,282	100.0%	277,071	100.0%	220,210	99.4%	17,128	95.7%
Yes	2106	0.4%	3	0.0%	85	0.0%	1243	0.6%	775	4.3%
Renal Failure										
No	566,311	99.9%	50,283	100.0%	277,127	100.0%	221,185	99.9%	17,716	99.0%
Yes	486	0.1%	2	0.0%	29	0.0%	268	0.1%	187	1.0%
Disseminated Cancer										
No	555,769	98.1%	50,211	99.9%	274,284	99.0%	214,086	96.7%	17,188	96.0%
Yes	11,028	1.9%	74	0.1%	2872	1.0%	7367	3.3%	715	4.0%
Smoker										
No	469,380	82.8%	46,060	91.6%	230,441	83.1%	179,155	80.9%	13,724	76.7%
Yes	97,417	17.2%	4225	8.4%	46,715	16.9%	42,298	19.1%	4179	23.3%
Wound Infection										
No	559,375	98.7%	50,094	99.6%	275,492	99.4%	217,014	98.0%	16,775	93.7%
Yes	7422	1.3%	191	0.4%	1664	0.6%	4439	2.0%	1128	6.3%
Weight Loss										
No	561,766	99.1%	50,214	99.9%	275,688	99.5%	218,311	98.6%	17,553	98.0%
Yes	5031	0.9%	71	0.1%	1468	0.5%	3142	1.4%	350	2.0%
Bleeding Disorder										
No	551,784	97.4%	50,199	99.8%	274,970	99.2%	210,950	95.3%	15,665	87.5%
Yes	15,013	2.6%	86	0.2%	2186	0.8%	10,503	4.7%	2238	12.5%
Transfusion										
No	566,322	99.9%	50,276	100.0%	277,015	99.9%	221,194	99.9%	17,837	99.6%
Yes	475	0.1%	9	0.0%	141	0.1%	259	0.1%	66	0.4%
Ventilator										
No	566,764	100.0%	50,284	100.0%	277,151	100.0%	221,433	100.0%	17,896	100.0%
Yes	33	0.0%	1	0.0%	5	0.0%	20	0.0%	7	0.0%

^a^*ASA-PS* American Society of Anesthesiologists Physical Status, ^b^*SIRS* Systemic inflammatory response syndrome, ^c^*COPD* Chronic obstructive pulmonary disease

**Table 3:** Surgery Characteristics of Study Sample

	All		ASA-PS 1		ASA-PS 2		ASA-PS 3		ASA-PS 4	
	no.	%	no.	%	no.	%	no.	%	no.	%
*n=*	566,797		50,285	8.9%	277,156	48.9%	221,453	39.1%	17,903	3.2%
CPT^a^ by Body System										
Digestive System	202,268	35.7%	17,597	35.0%	95,984	34.6%	83,510	37.7%	5177	28.9%
Musculoskeletal System	133,230	23.5%	13,518	26.9%	69,320	25.0%	48,197	21.8%	2195	12.3%
Integumentary System	55,656	9.8%	6837	13.6%	31,582	11.4%	16,422	7.4%	815	4.6%
Female Genital System	51,067	9.0%	6211	12.4%	32,055	11.6%	12,433	5.6%	368	2.1%
Cardiovascular System	33,281	5.9%	982	2.0%	4286	1.5%	21,381	9.7%	6632	37.0%
Nervous System	24,440	4.3%	1399	2.8%	12,114	4.4%	10,322	4.7%	605	3.4%
Urinary System	23,220	4.1%	824	1.6%	9606	3.5%	11,847	5.3%	943	5.3%
Endocrine System	20,993	3.7%	1294	2.6%	12,264	4.4%	7009	3.2%	426	2.4%
Male Genital System	10,173	1.8%	954	1.9%	5809	2.1%	3303	1.5%	107	0.6%
Respiratory System	6705	1.2%	65	0.1%	1460	0.5%	4684	2.1%	496	2.8%
Hemic/lymphatic System	3130	0.6%	118	0.2%	1392	0.5%	1549	0.7%	71	0.4%
Auditory System	1699	0.3%	442	0.9%	956	0.3%	291	0.1%	10	0.1%
Mediastinum/Diaphragm	821	0.1%	12	0.0%	250	0.1%	501	0.2%	58	0.3%
Maternity Care/Delivery	102	0.0%	24	0.0%	74	0.0%	4	0.0%	0	0.0%
Reproductive System/Intersex	12	0.0%	8	0.0%	4	0.0%	0	0.0%	0	0.0%
General	0	0.0%	0	0.0%	0	0.0%	0	0.0%	0	0.0%
Eye/Ocular Adnexa	0	0.0%	0	0.0%	0	0.0%	0	0.0%	0	0.0%
Top 10 CPT CCS^b^										
*Other*	165,715	29.2%	17,769	35.3%	69,833	25.2%	69,032	31.2%	9081	50.7%
Other hernia repair	45,057	7.9%	4579	9.1%	23,183	8.4%	16,444	7.4%	851	4.8%
Arthroplasty knee	39,155	6.9%	941	1.9%	19,391	7.0%	18,192	8.2%	631	3.5%
Hysterectomy, abdominal and vaginal	37,837	6.7%	3989	7.9%	24,025	8.7%	9532	4.3%	291	1.6%
Colorectal resection	32,851	5.8%	891	1.8%	15,974	5.8%	15,097	6.8%	889	5.0%
Inguinal and femoral hernia repair	26,881	4.7%	4960	9.9%	15,055	5.4%	6401	2.9%	465	2.6%
Cholecystectomy	26,553	4.7%	3282	6.5%	15,791	5.7%	7111	3.2%	369	2.1%
Gastric bypass	24,679	4.4%	110	0.2%	6890	2.5%	17,091	7.7%	588	3.3%
Hip replacement	24,094	4.3%	964	1.9%	13,123	4.7%	9556	4.3%	451	2.5%
Lumpectomy	22,100	3.9%	2774	5.5%	12,913	4.7%	6157	2.8%	256	1.4%

^a^*CPT* Current Procedural Terminology, ^b^Clinical Classification Software

### Development and concurrent validation of a model to predict ASA-PS

Additional file 1: Table S1 describes the details of our multinomial model with ASA-PS as an outcome. Across all ASA categories, conditions such as age, gender, race, or BMI were weakly predictive. However, conditions strongly predictive of ASA-PS status included total functional dependence, use of dialysis, insulin-dependent diabetes, disseminated cancer, COPD, hypertension treated with medications, and use of steroids.

Upon internal cross-validation, the overall C-statistic for the multinomial model (preoperative variables predicting observed ASA status) was C = 0.77 (95% CI: 0.766–0.773), signifying very good congruence between predicted and observed ASA status. The predicted ASA status agreed with the observed ASA-PS status for 99% of cases by one level (higher or lower) for ASA-PS 1–3 and for 85% for ASA-PS 4. In general, our model tended to upgrade observed ASA I’s to predicted ASA-PS II’s. At the same time the model tended to downgrade observed ASA-PS IV’s to predicted ASA-PS III’s and V’s to IV’s and III’s. This compression was most marked for observed ASA-PS IV. Preoperative factors associated with outliers for predicted ASA status revealed that in general discordances were found with extremes of age and BMI; procedures for musculoskeletal, nervous, and cardiovascular system CPT classes; and with diagnoses such as diabetes. In terms of concurrent validity, the group with predicted ASA-PS > observed ASA-PS had more comorbidities than patients with predicted ASA-PS < observed ASA-PS (mean 1.0 vs. 0.84, *p* < 0.001), higher BMI (30.7 vs. 29.6, *p* < 0.001), and a trend toward more functional limitations (98.95% independent vs. 99.03% independent, *p* = 0.067).

### Assessing the predictive validity of the ASA-PS model

**Fig. 2:**
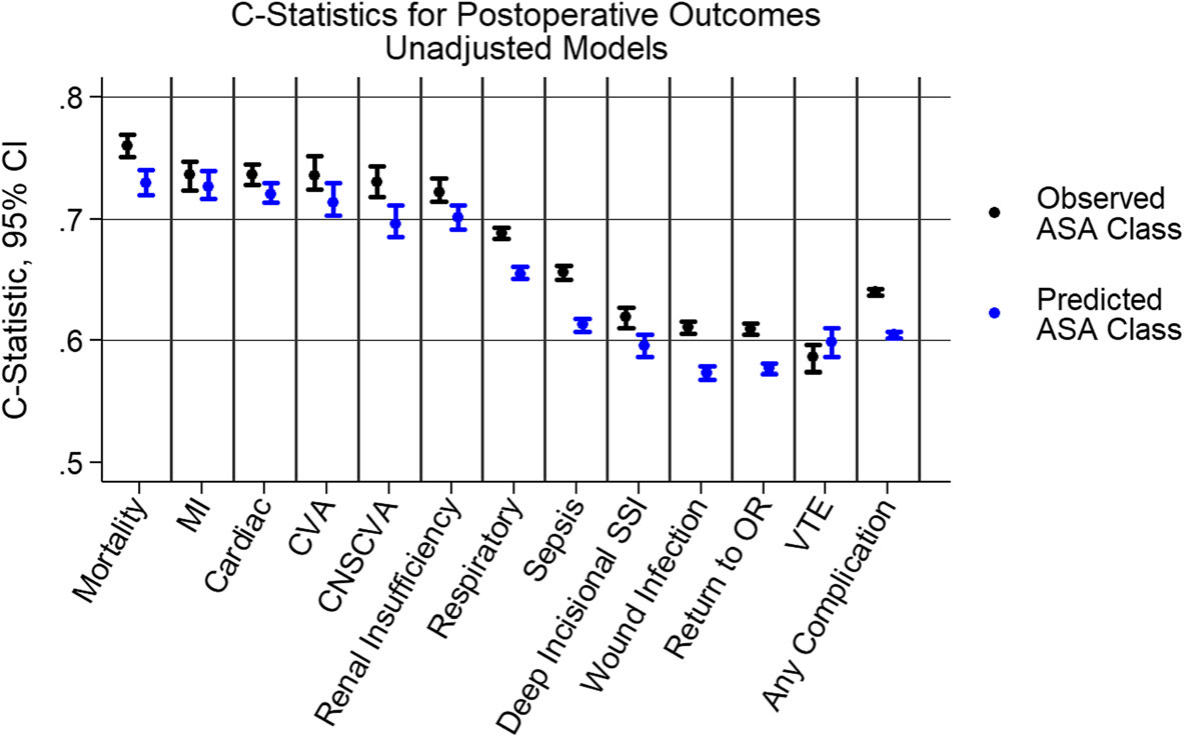
C-Statistics for Post-Operative Outcomes (Unadjusted)

**Fig. 3:**
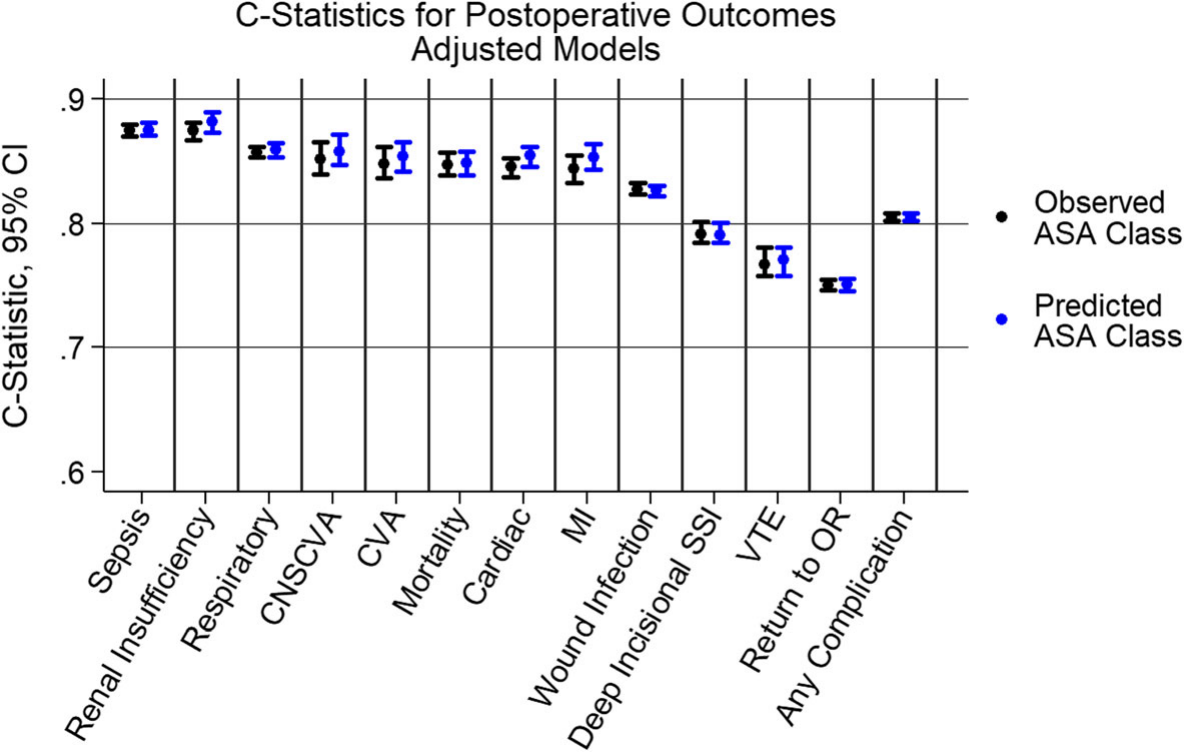
C-Statistics for Post-Operative Outcomes (Adjusted)

Using unadjusted, predicted ASA status to predict 30-day postoperative outcomes (predictive validity), the C-statistics ranged from 0.57–0.73 with mortality at 0.73 (95% CI: 0.719–0.740, Fig. 2). In comparison, using observed ASA status alone the C-statistics ranged from 0.58–0.76 with mortality at 0.73 (95% CI: 0.719–0.740). The multivariable models (that included adjustment factors such as anesthesia type and operation time) increased the ability of the models to discriminate for the various outcomes with covariates explaining some of the variance in outcomes (Fig. 3). Higher C-statistics were noted for individual complications (i.e.,sepsis,renal insufficiency) vs. the category of any complication. Patients with predicted ASA-PS > observed ASA-PS had a lower proportion of complications (3.4% vs 6.7%, *p* < 0.001) and lower mortality (0.13% vs 0.41%, *p* < 0.001).

**Online risk calculator:** The online calculator may be accessed at https://s-spire-clintools.shinyapps.io/ASA_PS_Estimator/

## Discussion

Our objective was to develop a model for predicting ASA-PS status using a wide range of preoperative predictors. We assessed both the concurrent and the predictive validity of predicted ASA-PS relative to observed ASA-PS and a variety of outcomes including 30-day morbidity and mortality. We noted that predicted ASA-PS was more closely associated with key indicators of preoperative status including comorbidities and higher BMI with a trend towards functional status compared to observed ASA-PS on. The overall accuracy (C = 0.77) of our model was comparable to measurements of inter-rater reliability found when anesthesiologists evaluated preoperative physical status [2, 24–27]. Our study further highlights the advantages and challenges of using entirely preoperative data for risk calculation, especially as surgical risk models continue to be built [7, 8].

The ability to have an electronically derived measure of ASA-PS can potentially assist with research, quality measurement, resource allocation, and clinical applications. Studies indicate that higher ASA-PS patients can experience increased morbidity and mortality, higher rates of hospital readmissions, and costs when undergoing ambulatory surgery procedures [28–30]. With the aim of lowering surgical risks and improving patient outcomes, ASA-PS is increasingly being used by government bodies and insurance agencies to justify the need for hospital inpatient admission either for pre- or post-operative management [31, 32]. For example, preoperative hospital admission may be necessary for optimization of comorbidities like congestive heart failure; a postoperative admission instead may be indicated to avoid physiologic deterioration or to maintain functional status in the setting of surgical trauma. While observed ASA-PS correlated more strongly with 30-day outcomes than predicted ASA-PS in our study, predicted ASA-PS values could be useful in situations where a pre-populated estimate in an EHR is needed (see Shiny application) or in studies where some or all cases are missing ASA-PS values [33]. Alignment of provider or facility decisions with government or payor guidelines can then be evaluated post-hoc for sample stratification where ASA-PS is missing and for quality monitoring. Calculation of a predicted ASA-PS could also aid in care coordination at the time of initial preoperative evaluation [5, 7, 14, 34]. Guidelines suggest that non-anesthesia clinicians when faced with the need to provide moderate or deep sedation for ASA-PS III or IV should have an anesthesia clinician present to avoid adverse outcomes such as respiratory arrest [35]. An automated process for calculating ASA-PS may therefore be helpful in generating electronic prompts and reminders.

Our study extends methods and results by Davenport et al. that evaluated the relationship between predicted ASA-PS, other preoperative risk factors, and 30- day morbidity and mortality outcomes [36]. While our study was a national level sample, Davenport’s study used National Surgical Quality Improvement Project data for 5878 surgical patients at a university medical center; they similarly noted that observed ASA-PS was a stronger predictor of 30-day outcomes than predicted ASA-PS. A potential explanation for these types of discordances is that ASA-PS remains a subjective measure of disease status and prone to differences in opinion when offered discrete categories (i.e.1,2). While the discrimination of our multivariable adjusted models in predicting 30-day outcomes was better than with univariate models, our results like Davenport’s suggest the further need for intraoperative variables and facility level data for risk adjustment. The fact that observed ASA-PS correlated more strongly with 30-day outcomes than predicted ASA –PS may reflect the fact that there are other confounding variables that still need to be accounted for in our predictive model. A prominent category of variables that we were unable to include was patients’ preoperative laboratory data; laboratory data was found to be not missing at random (NMAR) in our sample. In their predictive model for ASA-PS, Davenport et al. found that laboratory measures such as low serum albumin, high white blood cell count, and low hematocrit were strong predictors of 30-day outcomes [36]. The ability to include preoperative laboratory data could further strengthen the predictive validity of our model.

### Limitations and strengths

Given that our study involved the use of preexisting data, there are certain limitations and strengths. The analysis of preexisting data is always limited by its extant quality, the type of elements that it collects and the number of facilities. However, our study sample was a national cohort drawn from a broad variety of facilities across a wide geographic distribution. We also evaluated multiple sociodemographic criteria along with detailed clinical data and administrative data to enhance external validity. Another potential limitation is that while the ACS-NSQIP data set captures a wide variety of cases, nevertheless, it is still not 100% case capture. Nevertheless, our sample included a diverse set of procedures with detailed clinical data and administrative data that enhanced the concurrent validity of our models. As a national, clinical registry, the ACS-NSQIP is bound to contain some level of error or inconsistency. We attempted to exclude patients with a preoperative stay due to concerns that a preoperative stay might indicate the presence of conditions (like myocardial infarction) or events (need for preoperative optimization) that might enter into determination of ASA-PS. In addition, these conditions may not necessarily be observable through the ACS-NSQIP database. However, even with this exclusion, a very small number of patient with other indicators suggesting a preoperative stay, such as the 33 patients (out of the total sample of 566,797) who were on a ventilator in the 48 h prior to surgery, were not excluded. Given the very rare nature of these apparent errors or inconsistencies, we maintain that any potential bias would be negligible.

## Conclusions

The ability to have an electronically-derived measure of ASA-PS can assist with resource allocation and quality measurement as well as care coordination [33].. The model- based approach demonstrated here appears to be equal to or more valid than existing holistic judgments. An important key challenge is to demonstrate how these automatic approaches might be accepted by clinicians and actually add value. It has been widely published in the decision making literature that statistical models outperform crude judgments [37]. The original ASA-PS score, despite being a ‘quick-and-dirty’ tool, is easy to use and easy to apply. Approaches that combine subjective judgments and objective information may outperform both and should be evaluated as part of an implementation study. In addition, by enabling the prediction of ASA-PS when it is not available and potentially improving the measure’s accuracy when ASA-PS is available, we also see our effort as an aid for other researchers and clinicians to build better risk prediction models. Further external validation of this model with non-ACS-NSQIP data will help define the minimal number of elements needed to predict ASA-PS and further refine predictive ability [26, 38, 39].

## Supplementary information

**Additional file 1:** **Table S1.** Coefficients for the Multinomial Model to Predict ASA Class by Major Pre-operative Factors.

## Data Availability

Our data source was the 2014 Participant Use File (PUF) of the American College of Surgeons National Surgical Quality Improvement Program (ACS-NSQIP), which is available to member institutions.

## Abbreviations

ACS NSQIP: American College of Surgeons National Surgical Quality Improvement Program
ASA-PS: American Society of Anesthesiologists Physical Status Classification system
BMI: Body mass index
CCS: Clinical Classification Software
MAC: Monitored anesthesia care
PUF: Participant Use File
SIRS: Systemic inflammatory response syndrome
