# Supplementary material for: Development and validation of a predictive model for American Society of Anesthesiologists Physical Status
Source: BMC Health Serv Res. 2019 Nov 21;19:859. doi: 10.1186/s12913-019-4640-x (PMC6868867; doi:10.1186/s12913-019-4640-x)
Supplement: Supplementary file 1 — Additional file 1: Table S1. Coefficients for the Multinomial Model to Predict ASA Class by Major Pre-operative Factors. [file 12913_2019_4640_MOESM1_ESM.docx]

| **Additional file 1: Table S1: Coefficients for the Multinomial Model to Predict ASA Class by Major Pre-operative Factors** | | | | | | | | | |
| --- | --- | --- | --- | --- | --- | --- | --- | --- | --- |
| **Variable** | **ASA-PS 4** | | | **ASA-PS 3** | | | **ASA-PS 2** | | |
|  | **Coefficient** | **Z Score** | **p value** | **Coefficient** | **Z Score** | **p value** | **Coefficient** | **Z Score** | **p value** |
| (Intercept) | -13.9543 | -166.1995 | <0.001 | -8.8161 | -212.4986 | <0.001 | -3.2435 | -95.4129 | <0.001 |
| Age | 0.1136 | 127.8946 | <0.001 | 0.0888 | 193.1690 | <0.001 | 0.0498 | 127.0160 | <0.001 |
| ASCITESYes | 3.0893 | 38.8901 | <0.001 | 2.3714 | 45.4019 | <0.001 | 1.5180 | 22.1940 | <0.001 |
| BLEEDDISYes | 3.2398 | 27.2220 | <0.001 | 2.5997 | 22.3275 | <0.001 | 1.1742 | 10.1310 | <0.001 |
| BMI | 0.1594 | 101.8577 | <0.001 | 0.1623 | 150.4207 | <0.001 | 0.0884 | 88.5322 | <0.001 |
| DIABETESInsulin | 4.3576 | 261.4184 | <0.001 | 3.7736 | 335.4633 | <0.001 | 2.2752 | 152.8701 | <0.001 |
| DIABETESNon-Insulin | 2.5815 | 30.6190 | <0.001 | 2.4611 | 30.1903 | <0.001 | 1.7207 | 21.1617 | <0.001 |
| DIALYSISYes | 8.5346 | 235.2717 | <0.001 | 6.4183 | 195.7830 | <0.001 | 3.2429 | 54.6546 | <0.001 |
| DISCANCRYes | 3.9785 | 31.2325 | <0.001 | 3.6854 | 30.5017 | <0.001 | 2.2150 | 18.4266 | <0.001 |
| DYSPNEAAT REST | 2.7350 | 52.6613 | <0.001 | 1.7183 | 44.4305 | <0.001 | 0.7487 | 12.7572 | <0.001 |
| DYSPNEAMODERATE EXERTION | 2.3686 | 31.4688 | <0.001 | 1.7133 | 23.6788 | <0.001 | 0.8791 | 12.2560 | <0.001 |
| FNSTATUS2Partially Dependent | 3.1940 | 19.2804 | <0.001 | 2.6366 | 16.4347 | <0.001 | 1.4735 | 9.3061 | <0.001 |
| FNSTATUS2Totally Dependent | 13.6871 | 131.9365 | <0.001 | 12.3859 | 161.1794 | <0.001 | 9.3889 | 75.7694 | <0.001 |
| HXCHFYes | 3.3625 | 67.2081 | <0.001 | 2.1050 | 47.6050 | <0.001 | 0.1830 | 2.3195 | 0.0204 |
| HXCOPDYes | 3.7439 | 224.0144 | <0.001 | 3.0614 | 267.6368 | <0.001 | 1.6174 | 100.7293 | <0.001 |
| HYPERMEDYes | 3.4455 | 87.0076 | <0.001 | 3.0820 | 90.2404 | <0.001 | 2.4536 | 72.4941 | <0.001 |
| PRSEPISSepsis | 1.4149 | 10.9531 | <0.001 | 0.9581 | 10.2244 | <0.001 | 0.7236 | 6.3416 | <0.001 |
| PRSEPISSeptic Shock | -2.1732 | -18572 | <0.001 | -3.2229 | -28648 | <0.001 | -8.8261 | -64475734 | <0.001 |
| PRSEPISSIRS | 1.0858 | 6.5680 | <0.001 | 0.8043 | 6.0584 | <0.001 | 0.4915 | 4.0012 | <0.001 |
| RACE_ETHAsian | -1.0059 | -12.6879 | <0.001 | -0.5335 | -15.5880 | <0.001 | -0.3188 | -11.2173 | <0.001 |
| RACE_ETHBlack | 0.0759 | 2.1429 | 0.0321 | 0.0139 | 0.5886 | 0.5561 | -0.0811 | -3.7493 | <0.001 |
| RACE_ETHHispanic | -0.2667 | -6.2701 | <0.001 | -0.3095 | -13.5547 | <0.001 | -0.2213 | -11.1971 | <0.001 |
| RACE_ETHOther | -0.3269 | -2.7317 | 0.0063 | 0.0318 | 0.4905 | 0.6238 | -0.0828 | -1.4050 | 0.1600 |
| RACE_ETHUnknown | -0.9651 | -32.9956 | <0.001 | -1.0968 | -69.0318 | <0.001 | -0.7472 | -55.5255 | <0.001 |
| RENAFAILYes | 2.9313 | 28.9330 | <0.001 | 2.2961 | 27.2377 | <0.001 | 0.7600 | 5.0118 | <0.001 |
| SEXMale | 0.1477 | 7.2837 | <0.001 | -0.3324 | -26.8063 | <0.001 | -0.5034 | -45.2016 | <0.001 |
| SMOKEYes | 2.0852 | 74.2974 | <0.001 | 1.8008 | 91.6814 | <0.001 | 1.2912 | 70.7389 | <0.001 |
| STEROIDYes | 3.4993 | 40.1583 | <0.001 | 3.2326 | 39.6427 | <0.001 | 2.2722 | 28.2628 | <0.001 |
| TRANSFUSYes | 2.9339 | 28.9296 | <0.001 | 2.1046 | 30.0221 | <0.001 | 1.2417 | 13.4366 | <0.001 |
| VENTILATYes | 2.4064 | 1147.7802 | <0.001 | 1.6508 | 1254.6483 | <0.001 | 0.4654 | 321.2113 | <0.001 |
| WNDINFYes | 1.8407 | 19.4597 | <0.001 | 1.2722 | 14.5513 | <0.001 | 0.4446 | 5.2609 | <0.001 |
| WTLOSSYes | 2.7460 | 19.2484 | <0.001 | 2.6312 | 20.1741 | <0.001 | 1.5357 | 11.9701 | <0.001 |
|  |  |  |  |  |  |  |  |  |  |
